# Proportion of children meeting recommendations for 24-hour movement guidelines and associations with adiposity in a 12-country study

**DOI:** 10.1186/s12966-016-0449-8

**Published:** 2016-11-25

**Authors:** Blanca Roman-Viñas, Jean-Philippe Chaput, Peter T. Katzmarzyk, Mikael Fogelholm, Estelle V. Lambert, Carol Maher, Jose Maia, Timothy Olds, Vincent Onywera, Olga L. Sarmiento, Martyn Standage, Catrine Tudor-Locke, Mark S. Tremblay, for the ISCOLE Research Group

**Affiliations:** 1 Department of Physical Activity and Sport Sciences, Psychology, Education and Sport Sciences, FPCEE Blanquerna, Universitat Ramon Llull, 34 Cister, 08022 Barcelona, Spain; 2 Nutrition Research Foundation, 242 Rocafort, 08029 Barcelona, Spain; 3 CIBER Fisiopatología de la Obesidad y Nutrición (CIBERobn), Instituto de Salud Carlos III (ISCIII), Madrid, Spain; 4 Children’s Hospital of Eastern Ontario Research Institute, 401 Smyth Road, Ottawa, ON K1H 8L1 Canada; 5 Pennington Biomedical Research Center, 6400 Perkins Road, Baton Rouge, LA 70808 USA; 6 Department of Food and Environmental Sciences, University of Helsinki, 00014 Helsinki, Finland; 7 Division of Exercise Science and Sports Medicine, Department of Human Biology, Faculty of Health Sciences, University of Cape Town, PO Box 115, Newlands, Cape Town South Africa; 8 Alliance for Research in Exercise Nutrition and Activity (ARENA), School of Health Sciences, University of South Australia, City East Campus, North Terrace, Adelaide 5000 Australia; 9 CIFI2D, Faculdade de Desporto, University of Porto, Porto, Portugal; 10 Department of Recreation Management and Exercise Science, Kenyatta University, Nairobi, Kenya; 11 School of Medicine Universidad de los Andes, Carrera 1ª N° 18A 12, Bogotá, Colombia; 12 Department for Health, University of Bath, Claverton Down, Bath BA2 7AY UK; 13 Department of Kinesiology, University of Massachusetts, 111 Totman, 30 Eastman Lane, Amherst, MA 01002 USA

**Keywords:** Physical activity, Screen time, Sleep, Recommendations, Obesity, Children, Prevalence

## Abstract

**Background:**

The Canadian 24-h movement guidelines were developed with the hope of improving health and future health outcomes in children and youth. The purpose of this study was to evaluate adherence to the 3 recommendations most strongly associated with health outcomes in new 24-h movement guidelines and their relationship with adiposity (obesity and body mass index z-score) across countries participating in the International Study of Childhood Obesity, Lifestyle and the Environment (ISCOLE).

**Methods:**

Cross-sectional results were based on 6128 children aged 9–11 years from the 12 countries of ISCOLE. Sleep duration and moderate-to-vigorous physical activity (MVPA) were assessed using accelerometry. Screen time was measured through self-report. Body weight and height were measured. Body mass index (BMI, kg · m^−2^) was calculated, and BMI z-scores were computed using age- and sex-specific reference data from the World Health Organization. Obesity was defined as a BMI z-score > +2 SD. Meeting the overall 24-h movement guidelines was defined as: 9 to 11 h/night of sleep, ≤2 h/day of screen time, and at least 60 min/day of MVPA. Age, sex, highest parental education and unhealthy diet pattern score were included as covariates in statistical models. Associations between meeting vs. not meeting each single recommendation (and combinations) with obesity were assessed with odds ratios calculated using generalized linear mixed models. A linear mixed model was used to examine the differences in BMI z-scores between children meeting vs. not meeting the different combinations of recommendations.

**Results:**

The global prevalence of children meeting the overall recommendations (all three behaviors) was 7%, with children from Australia and Canada showing the highest adherence (15%). Children meeting the three recommendations had lower odds ratios for obesity compared to those meeting none of the recommendations (OR = 0.28, 95% CI 0.18–0.45). Compared to not meeting the 24-h movement recommendations either independently or combined, meeting them was significantly associated with a lower BMI z-score. Whenever the MVPA recommendation was included in the analysis the odds ratios for obesity were lower.

**Conclusions:**

For ISCOLE participants meeting these 3 healthy movement recommendations the odds ratios of being obese or having high BMI z-scores were lower. However, only a small percentage of children met all recommendations. Future efforts should aim to find promising ways to increase daily physical activity, reduce screen time, and ensure an adequate night’s sleep in children.

**Trial registration:**

The International Study of Childhood Obesity, Lifestyle and the Environment (ISCOLE) was registered at ClinicalTrials.gov (Identifier NCT01722500) (October 29, 2012).

## Background

Physical activity guidelines for children and adolescents have traditionally focused on recommendations to achieve a certain quantity of physical activity at defined intensities (e.g., 60 min of moderate to vigorous physical activity (MVPA)) [1]. However, adhering to such guidelines accounts for a low proportion of the overall 24-h period [2], and does not consider other movement behaviors also associated with health and obesity, such as screen time and sleeping habits [3]. The 24-h movement guidelines recently developed in Canada represent a paradigm shift in thinking about movement behaviors [4] from a focus on a single specific movement behavior type (e.g., MVPA) to an integrated movement behavior model. They include recommendations for the 24-h period, taking into account those behaviors related to health outcomes, not only MVPA but also recreational screen time (no more than 2 h per day) and adequate sleep duration (e.g., between 9 and 11 h per night for children aged 5–13 years).

Isolated adherence to MVPA, screen time and sleep duration recommendations have been associated with lower adiposity indicators in children and adolescents [3, 5–10]. How combinations of these movements behaviors are associated with obesity is largely unknown as few studies have examined the influence of meeting specific movement behavior recommendation combinations and obesity, and only a subset have considered sleep duration as a health-related risk behavior in combined analyses [11–18]. In addition, most of the studies have been conducted in developed countries and only a few have included a multi-country analysis [16–18]. Only the International Study of Childhood Obesity, Lifestyle and the Environment (ISCOLE) has assessed MVPA, screen time and sleep duration in relation to obesity in countries with a wide range of economic level of development [19–21].

Hence, the objective of this study was to determine the proportion of participants who meet the MVPA, screen time, and sleep duration recommendations (and combinations of these recommendations) across the 12 countries participating in ISCOLE and evaluate the associations with obesity and body mass index (BMI) z-scores.

## Methods

### Setting

ISCOLE is a cross-sectional, multinational study designed to determine the relationships between lifestyle behaviors and obesity in 12 study sites located in Australia, Brazil, Canada, China, Colombia, Finland, India, Kenya, Portugal, South Africa, the United Kingdom and the United States. These countries represent a wide range of economic development (low to high income), Human Development Index (0.509 in Kenya to 0.929 in Australia) and inequality (GINI coefficient). The design and methods have been published in detail elsewhere [22]. By design, the within-site samples were not intended to be nationally representative. Rather, the primary sampling frame was schools, which were typically stratified by an indicator of socioeconomic status to maximize variability within sites. A standard protocol was used to collect data across all sites, and all study personnel underwent rigorous training and certification before and during the data collection to ensure the quality of data collected [22]. The Institutional Review Board at the Pennington Biomedical Research Center in Baton Rouge, USA (coordinating center) approved the ISCOLE protocol, and the Ethical Review Boards at each participating institution also approved the local protocol. Written informed consent was obtained from parents or legal guardians, and child assent was also obtained as required by local Ethical Review Boards before participation in the study. Data were collected from September 2011 to December 2013.

### Participants

The sample included 9–11 year-old children from the 12 ISCOLE sites. The recruitment goal was to enroll at least 500 children per site. A total of 7372 children participated in ISCOLE, of which 6128 remained in the present analytic dataset after excluding participants without valid accelerometry (*n* = 1214), information on screen time (*n* = 25) or BMI (*n* = 5). Participants excluded due to missing data had significantly higher BMI z-scores (0.61) compared to those included in the present analytical sample (0.46).

### Ascertainment of obesity

Body weight and height were measured according to standardized procedures by trained ISCOLE staff [22]. BMI (kg · m^−2^) was calculated, and BMI z-scores were computed using age- and sex-specific reference data from the World Health Organization (WHO) [23]. Participants were classified as obese (BMI z-score > +2 SD) or nonobese (BMI z-score ≤ +2 SD).

### Measurement of sleep duration and lifestyle behaviors

MVPA was objectively assessed using 24-h, waist-worn accelerometry. An Actigraph GT3X+ accelerometer (ActiGraph LLC, Pensacola, FL, US) was worn at the waist on an elasticized belt at the right mid-axillary line. Participants were encouraged to wear the accelerometer 24 h per day (removing only for water-related activities) for at least 7 days, including 2 weekend days. The minimal amount of monitored waking wear time that was considered acceptable for inclusion in the sample was at least 4 days with at least 10 h per day, including at least 1 weekend day. Data were collected at a sampling rate of 80 Hz, downloaded in 1-s epochs with the low frequency extension filter using the ActiLife software version 5.6 or higher (ActiGraph LLC, Pensacola, FL, U.S.A). Data were later reintegrated to 15-s and 60-s epochs for the different analyses. Nocturnal sleep duration was estimated from the accelerometry data using 60-s epochs and a fully automated algorithm for 24-h waist-worn accelerometers that was recently validated for ISCOLE [24]. This new algorithm produces more precise estimates of sleep duration than previous algorithms and captures total sleep time from sleep onset to the end of sleep, including all epochs and wakefulness after onset [24, 25]. The weekly total sleep time averages were calculated using only days where valid sleep was accumulated (total sleep period time ≥160 min) and only for participants with at least 3 nights of valid sleep, including 1 weekend night (Friday or Saturday). After exclusion of total sleep time and awake non-wear time (any sequence of ≥20 consecutive minutes of 0 activity counts), MVPA was defined as all activity ≥574 counts per 15 s [26]. After testing for normality, MVPA was log-transformed for analysis. Child-reported screen time was determined from a lifestyle questionnaire [22] and questions were obtained from the US Youth Risk Behavior Surveillance System [27]. Children were asked how many hours they typically watched TV, and how many hours they played video games and/or used the computer per week day, and per weekend day. Response options were 0, <1, 1, 2, 3, 4 and 5 or more hours per day. A daily average score was computed by recording ‘<1’ to ‘0.5’ and ‘5 or more hours’ to ‘5’, and weighting the responses (2/7 for weekend; 5/7 for weekday). Although not tested individually in every participating country, the TV viewing time question was shown to have adequate reliability with a one week test-retest interval (Spearman correlation = 0.55–0.68) and validity as compared to 7-day TV time use logs (Spearman correlation = 0.47) [28]. Furthermore, self-report methods of quantifying screen time have been reported to have acceptable reliability and validity in children [29].

### Covariates

Age, sex, highest parental education and unhealthy diet pattern score were included as covariates in statistical models. Age was computed from birthdates and measurement dates, and sex was recorded on a questionnaire. Overall, 594 participants (10%) were missing data on household income so education was used instead as a proxy for socioeconomic status. The highest level of parental education (with options ranging from less than high school to graduate degree) was reported by the parent or guardian and three categories were created to facilitate analysis across sites (did not complete high school, completed high school or some college, and bachelor’s or postgraduate degree). Dietary patterns of children were assessed using a 23-item food frequency questionnaire, and principal components analyses were carried out using weekly portions as input variables [30, 31]. The “unhealthy diet pattern” was characterized by a high consumption of fast foods, ice cream, fried food, French fries, potato chips, cakes and sugar-sweetened sodas, and was included as a covariate in this paper. Of note, biological maturity was estimated using the maturity offset method in ISCOLE; however, because age and weight are included in the maturity offset calculation, biological maturity was not included as a covariate in our analyses.

### Statistical analysis

Statistical analyses were conducted using SAS version 9.4 and JMP version 12 (SAS Institute, Cary, NC, US). Means and standard deviations of descriptive characteristics were computed by study site. We also calculated the proportion of children meeting the weekly average MVPA (≥60 min/day), screen time (≤2 h/day), and sleep duration (9–11 h/night) [4, 32] recommendations, and combinations of these recommendations, by study site. Associations between meeting vs. not meeting these recommendations, alone or in combinations, with obesity (0, no; 1, yes) were assessed with odds ratios calculated using generalized linear mixed models (PROC GLMMIX). Study sites were considered to have fixed effects, and schools nested within study sites were viewed as having random effects. The denominator degrees of freedom for statistical tests pertaining to fixed effects were calculated using the Kenward and Roger approximation [33]. Age, sex, highest parental education and unhealthy diet pattern score were included as covariates in the models. A subsequent linear mixed model (PROC MIXED) was used to examine the differences in BMI z-scores between children meeting vs. not meeting the different combinations of recommendations. Models were presented for the total sample and by sex only; analyses by study site were not possible due to a lack of statistical power for certain combinations of movement behaviors. The level of significance was set at *P* < 0.05.

## Results

Table 1 shows the descriptive characteristics of participants by study site. The mean overall MVPA time was 60 min/day and ranged from 45 min/day in China to 72 min/day in Kenya. The mean screen time was 2.9 h/day and ranged from 2 h/day in India to 3.4 h/day in the USA. The mean sleep duration was 8.8 h/day in the overall sample, highest in the UK (9.5 h/day) and lowest in Portugal (8.3 h/day). The overall prevalence of obesity was 12.3% and ranged from 5.4% in Finland to 24.5% in China.

**Table 1:** Descriptive characteristics of participants stratified by study site (*n* = 6128)

Country (site)	Participants (n, % males)	Age (years)	MVPA (min/day)	Screen time (h/day)	Sleep duration (h/day)	BMI (kg/m^2^)	Obesity^a^ (%)
Australia (Adelaide)	451 (46.3)	10.8 (0.5)	65.4 (23.1)	3.0 (1.6)	9.4 (0.7)	18.8 (3.2)	10.4
Brazil (Sao Paulo)	469 (48.6)	10.5 (0.5)	59.6 (26.3)	3.9 (2.2)	8.6 (0.8)	19.7 (4.4)	21.5
Canada (Ottawa)	507 (41.0)	10.5 (0.4)	58.4 (19.4)	2.8 (1.8)	9.1 (0.8)	18.3 (3.4)	12.0
China (Tianjin)	465 (51.4)	9.9 (0.5)	44.7 (15.7)	2.2 (1.5)	8.8 (0.6)	18.9 (4.2)	24.5
Colombia (Bogotá)	822 (49.0)	10.5 (0.6)	68.2 (24.9)	3.0 (1.5)	8.8 (0.8)	17.6 (2.5)	5.6
Finland (Helsinki, Espoo and Vantaa)	461 (45.3)	10.5 (0.4)	70.1 (26.8)	3.0 (1.5)	8.5 (0.9)	17.8 (2.6)	5.4
India (Bangalore)	532 (45.3)	10.5 (0.5)	48.9 (21.2)	2.0 (1.2)	8.6 (0.7)	17.9 (3.3)	10.7
Kenya (Nairobi)	459 (45.3)	10.3 (0.7)	72.0 (31.3)	2.5 (1.7)	8.6 (0.9)	17.2 (3.2)	6.8
Portugal (Porto)	639 (42.9)	10.5 (0.3)	55.7 (21.5)	2.5 (1.4)	8.3 (0.9)	19.4 (3.4)	16.4
South Africa (Cape Town)	453 (39.1)	10.3 (0.7)	64.9 (25.5)	3.3 (2.0)	9.2 (0.7)	18.0 (3.6)	10.8
UK (Bath and North East Somerset)	414 (43.7)	10.9 (0.5)	63.8 (22.9)	3.2 (1.6)	9.5 (0.7)	18.5 (2.9)	8.9
USA (Baton Rouge)	456 (40.8)	10.0 (0.6)	50.1 (18.9)	3.4 (2.2)	8.9 (0.9)	18.8 (3.8)	17.5
All sites	6128 (45.1)	10.4 (0.6)	60.3 (24.9)	2.9 (1.8)	8.8 (0.9)	18.4 (3.5)	12.3

Abbreviations: *MVPA* moderate-to-vigorous physical activity, *BMI* body mass index. Data are shown as mean (standard deviation) unless otherwise indicated

^a^Obesity defined according to the World Health Organization criteria [23]

MVPA, and sleep duration were based on accelerometer data, screen time was self-reported

Table 2 shows the proportion of individuals meeting the 24-h movement guidelines by study site. Nineteen percent of the overall sample met none of the recommendations. Brazil, Portugal and the USA (29% each) were the countries with the highest prevalence of adherence to none of the recommendations, and Australia showed the lowest percentage of non-adherence (7%). The mean overall adherence to the MVPA, screen time and sleep time recommendations were 44, 39 and 42%, respectively. The population with highest adherence to the MVPA guideline was found in Finland (61%) and the population with the lowest adherence was found in China (15%). The highest and lowest proportion of the population meeting the screen time guideline was found in India (62%) and Brazil (24%), respectively. The highest adherence to the sleep time recommendation was found in Australia and the UK (76%), and the lowest was found in Portugal (18%). Regarding the adherence to the 24-h movement guidelines, the countries with the highest prevalence to the overall recommendations were Australia and Canada (15 and 14% of the sample, respectively); the lowest adherence was found in China (2%).

**Table 2:** Proportion of participants meeting the MVPA, screen time, and sleep duration recommendations and combinations of these recommendations by study site

Country (site)	None (%)	MVPA (%)	ST (%)	SLEEP (%)	MVPA + ST (%)	MVPA + SLEEP (%)	ST + SLEEP (%)	MVPA + ST + SLEEP (%)
Australia (Adelaide)	7.1	55.4	35.3	75.8	19.7	41.5	27.3	14.9
Brazil (Sao Paulo)	28.8	43.9	23.9	29.9	11.3	9.8	8.7	3.4
Canada (Ottawa)	14.4	42.6	44.8	58.6	20.3	24.7	29.4	14.0
China (Tianjin)	21.1	15.1	59.1	34.2	6.2	4.9	19.8	1.5
Colombia (Bogotá)	16.8	59.5	31.8	37.6	19.2	21.1	13.0	7.8
Finland (Helsinki, Espoo and Vantaa)	19.1	61.4	33.0	28.2	20.8	15.6	11.9	6.7
India (Bangalore)	22.4	25.0	62.0	26.9	16.5	8.0	17.7	6.0
Kenya (Nairobi)	16.3	58.1	42.2	31.3	27.2	16.6	10.9	6.5
Portugal (Porto)	28.5	35.1	45.4	18.1	15.8	4.1	9.2	2.0
South Africa (Cape Town)	13.0	51.7	34.0	60.7	17.7	32.7	21.0	11.9
UK (Bath and North East Somerset)	8.0	50.7	26.8	75.8	14.5	36.5	21.7	11.4
USA (Baton Rouge)	28.5	26.5	32.0	43.0	7.2	9.9	15.1	2.1
All sites	19.0	44.1	39.3	41.9	16.6	18.2	16.7	7.2

Meeting the recommendations is defined as ≥60 min/day for MVPA, ≤2 h/day for screen time, and between 9 and 11 h/night for sleep duration

MVPA and sleep duration were based on accelerometer-determined while screen time was self-reported

*MVPA* moderate-to-vigorous physical activity, *ST* screen time, *SLEEP* sleep duration

**Fig. 1:**
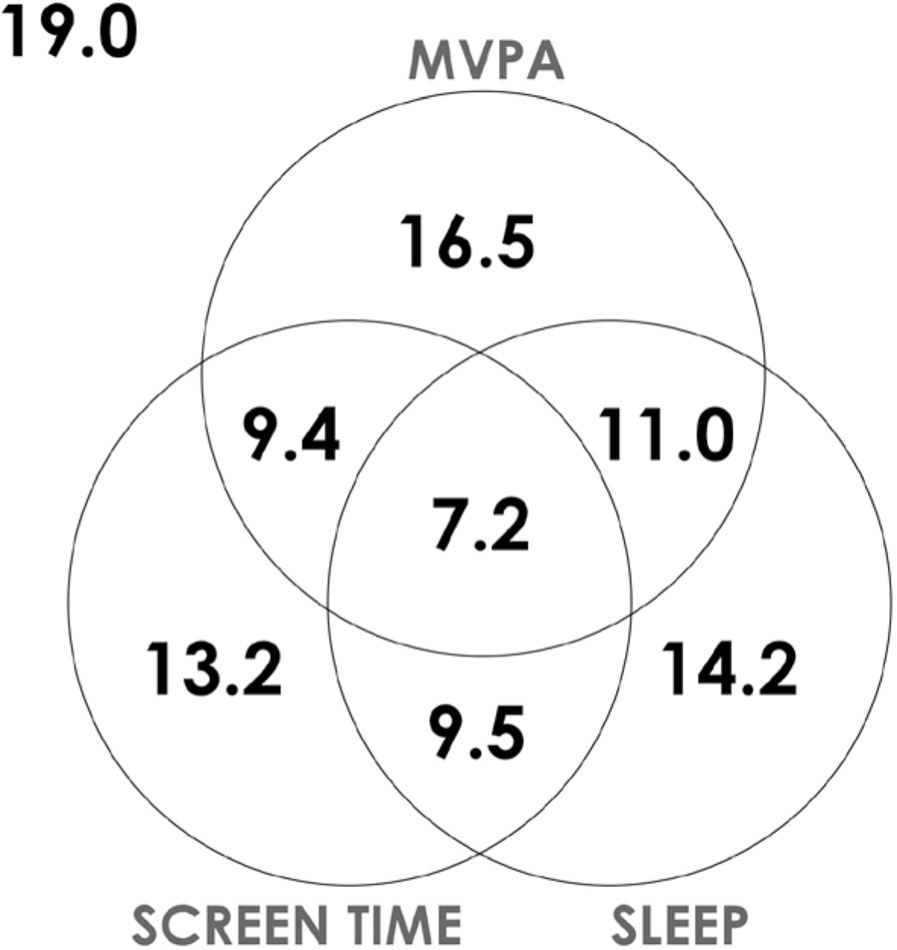
Venn diagram showing the proportion (%) of participants meeting no guidelines, the moderate-to-vigorous physical activity (MVPA), screen time, and sleep duration recommendations, and combinations of these recommendations in the full study sample (*n* = 6128)

Figure 1 illustrates, the proportion of participants meeting the MVPA, screen time, and sleep duration recommendations, and combinations of these recommendations, in the full study sample. The figure clearly shows that there is very little co-occurrence of movement behaviors in this sample of children.

The odds ratios for the independent and combined associations of meeting vs. not meeting the MVPA, screen time and sleep duration recommendations with obesity are shown in Table 3. Children who meet the three recommendations are 72% less likely to be obese than those who do not meet them. Adherence to any combination of two recommendations resulted in lower odds ratios of being obese compared to children not meeting the recommendations. Except for the screen time recommendation in boys and girls separately, the adherence to a single recommendation resulted in a lower odds ratio of being obese, especially when adhering to the MVPA recommendation.

**Table 3:** Odds ratios for obesity^a^ associated with meeting vs. not meeting MVPA, screen time, and sleep duration recommendations and combinations of these recommendations in the full study sample

	Total sample (*n* = 6128)		Boys (*n* = 2763)		Girls (*n* = 3365)	
	OR	95% CI	OR	95% CI	OR	95% CI
Meeting the following recommendation:						
MVPA	0.45*	0.38–0.52	0.42*	0.35–0.50	0.28*	0.20–0.38
ST	0.86*	0.75–0.98	0.95	0.79–1.14	0.86	0.70–1.05
SLEEP	0.67*	0.58–0.77	0.69*	0.57–0.84	0.66*	0.53–0.82
MVPA + ST	0.43*	0.33–0.55	0.53*	0.40–0.69	0.20*	0.11–0.34
MVPA + SLEEP	0.38*	0.29–0.49	0.41*	0.30–0.54	0.22*	0.13–0.38
ST + SLEEP	0.65*	0.52–0.80	0.74*	0.55–0.98	0.61*	0.44–0.83
All three recommendations	0.28*	0.18–0.45	0.38*	0.22–0.64	0.11*	0.04–0.35

Models adjusted for age, sex (combined analysis), highest parental education and unhealthy diet pattern score

Meeting the recommendations is defined as ≥60 min/day for MVPA, ≤2 h/day for screen time, and between 9 and 11 h/night for sleep duration

MVPA and sleep duration were accelerometer-determined while screen time was self-reported

*MVPA* moderate-to-vigorous physical activity, *ST* screen time, *SLEEP* sleep duration, *OR* odds ratio, *CI* confidence interval

^a^Obesity defined according to the World Health Organization criteria [23]

*P < 0.05

Table 4 shows the mean BMI z-scores in children meeting and not meeting the MVPA, screen time and sleep duration recommendations, and the combinations of recommendations in the overall sample. Children meeting individual recommendations, two recommendations or all three of the recommendations had a significantly lower BMI z-score than children not meeting any recommendations. Meeting the 24-h guidelines was associated with having the lowest BMI z-score in boys (0.07) and girls (−0.05). When meeting only one of the recommendations, the adherence to the MVPA recommendation was the one that was associated with the lowest BMI z-score.

**Table 4:** Differences in BMI z-score between children meeting vs. not meeting the MVPA, screen time, and sleep duration recommendations and combinations of these recommendations in the full study sample (*n* = 6128)^1^

	Total sample (*n* = 6128)		Boys (*n* = 2763)		Girls (*n* = 3365)	
	BMI z-score	95% CI	BMI z-score	95% CI	BMI z-score	95% CI
MVPA						
Meet	0.24*	0.20–0.29	0.33*	0.27–0.39	0.09*	0.02–0.15
Do not meet	0.63	0.58–0.67	0.89	0.80–0.97	0.51	0.45–0.56
ST						
Meet	0.36*	0.31–0.41	0.44*	0.35–0.53	0.31*	0.25–0.37
Do not meet	0.53	0.49–0.57	0.61	0.55–0.67	0.45	0.39–0.51
SLEEP						
Meet	0.34*	0.29–0.38	0.42*	0.34–0.50	0.29*	0.22–0.35
Do not meet	0.55	0.51–0.59	0.64	0.57–0.70	0.48	0.42–0.53
MVPA + ST						
Meet	0.12*	0.05–0.19	0.17*	0.07–0.28	0.05*	–0.04–0.14
Do not meet	0.53	0.49–0.57	0.65	0.59–0.71	0.43	0.38–0.48
MVPA + SLEEP						
Meet	0.12*	0.05–0.18	0.21*	0.11–0.30	−0.01*	–0.10–0.09
Do not meet	0.53	0.49–0.57	0.65	0.58–0.70	0.44	0.39–0.49
ST + SLEEP						
Meet	0.23*	0.15–0.30	0.32*	0.19–0.45	0.18*	0.08–0.27
Do not meet	0.50	0.46–0.53	0.58	0.52–0.63	0.43	0.38–0.48
All three recommendations						
Meet	0.01*	−0.09–0.11	0.07*	−0.08–0.23	−0.05*	−0.17–0.07
Do not meet	0.49	0.45–0.52	0.58	0.53–0.64	0.41	0.36–0.45

Meeting the recommendations is defined as ≥60 min/day for MVPA, ≤2 h/day for screen time, and between 9 and 11 h/night for sleep duration

Multilevel models are adjusted for age, sex (combined analysis), highest parental education and unhealthy diet pattern score

Values are least square means (SEM)

MVPA and sleep duration were accelerometer-determined while screen time was self-reported

*MVPA* moderate-to-vigorous physical activity, *ST* screen time, *SLEEP* sleep duration, *BMI* body mass index, *CI* confidence interval

*P < 0.05 vs. do not meet the recommendation

^1^BMI z-score determined according to World Health Organization criteria [23]

## Discussion

This is the first study showing that adherence to 3 key healthy behaviors of the 24-h movement guidelines (defined as at least 60 min per day of MVPA, no more than 2 h per day spent on recreational screen time, and a sleep duration between 9 and 11 h per night) (4) was associated with lower odds ratios for obesity in an international sample of children. The probability of being obese when meeting the three recommendations was the lowest compared to meeting none, one, or a combination of two recommendations. In the overall sample, the lower odds ratios for obesity increased when any additional recommendation was added to a single one. In other words, other movement behaviors across the whole day seem to matter (not only MVPA). However, the MVPA recommendation was the single recommendation associated with the lowest overall odds ratios for obesity. Finally, children meeting one, two, or three guideline recommendations had significantly lower BMI z-scores compared to those not meeting them.

The relationship of adherence to the 24-h movement guidelines with obesity had been previously evaluated in a sample of Canadian and US children and adolescents [12–14]. The authors found that boys and girls not meeting the 3 movement behavior recommendations were more likely to be obese than those meeting them. Moreover, not meeting the MVPA recommendation was the strongest predictor for being obese regardless of screen time and sleeping time [12–14]. Our study also showed that children not meeting the MVPA guideline showed the highest odds ratios for obesity. This relationship with MVPA was more evident in girls, possibly due to the fact that girls spent less time in MVPA [19].

Other studies have evaluated the influence of energy-related behaviors (including data on diet or some food group’s intake or breakfast consumption, physical activity, sedentary time and sleep duration) and obesity in children and adolescents [15–17, 34, 35]. The studies by Duncan et al. [34] and Danielzik et al. [35] were the first ones that examined the cumulative effects of multiple risk factors on adiposity in children. Except for the study by Duncan et al. that was conducted in New Zealand [34], all of them were conducted in European countries, either analyzing selected samples [35], national samples [15] or multi-country data [16, 17]. Pérez-Rodrigo et al. [15] identified a combination of high physical activity level, low sedentary time and longer sleep time to be associated with a lower prevalence of obesity in a representative sample of Spanish children. An unhealthier combination of low physical activity and poor diet was also more prevalent in girls, older children and those from lower socio-economic backgrounds [15]. The ENERGY study (EuropeaN Energy balance Research to prevent excessive weight Gain among Youth) included data from Belgium, Greece, Hungary, the Netherlands, Norway, Slovenia and Spain [16]. They identified a short sleeper inactive cluster that was most prevalent in Greece and contained the highest proportion of overweight and obese boys and girls. Finally, the study by Wijnhoven et al. [17] contained data from the European Childhood Obesity Surveillance Initiative (COSI) and evaluated the relationship between some health risk behaviors related to diet and physical activity and overweight and obesity in children from Bulgaria, Czech Republic, Lithuania, Portugal and Sweden. They found that children with an unhealthier physical activity-risk behavior score (defined as the combination of the use of inactive transportation going to and from school, going to a sports or dancing club less than 2 days/week, playing outside less than 1 h/day, engaging in screen time 2 or more hours/day, and a sleep duration lower than 9 h/day) were more likely to be obese than children with a better score.

In comparison to the studies mentioned above, the countries participating in ISCOLE have a much wider range of economic and social development levels [20, 22], which increases and extends the knowledge about how the obesity determinants are affected by social, cultural and geographic factors. Although it was not possible to evaluate the relationship between the combined guideline recommendations and obesity by country site due to the small sample size adhering to the overall guidelines, there was a trend showing similar results across all the countries in the study (data not shown), stressing the importance of the 24-h movement recommendations to prevent obesity in all countries.

It is concerning to see very low levels of adherence to the combined recommendations, especially in sites such as China, Portugal, the US and Brazil, which are the sites with the highest prevalence of obesity in ISCOLE. The results are similar to those found in the WHO analysis [17] where only 8% of children achieved the best classification in the physical activity-risk behavior score and the Canadian and US studies, with a range of compliance between 5 and 17% of the population [12–14]. When looking at the individual movement behavior recommendations, less than 50% of children in most of the ISCOLE countries met the recommendations, especially for the sleep time and screen time. A low proportion of children from China, India and the USA met the recommendation of being physically active at least 60 min per day, data in line with the 15 to 28% prevalence coming from the International Children’s Accelerometry database [36]. In addition to not meeting the minimum amount of recommended physical activity, the majority of the sample engaged in recreational screen time for more than 2 hours per day, behaviors most frequently related to negative health outcomes [8, 9].

How children distribute their time during the day may affect their total energy expenditure. MVPA only accounts for a small proportion (<5%) of the 24-h period, with the remaining 95% dedicated to sleep, sedentary behavior and light-intensity activities [2]. Although there is no clear consensus about how to define light-intensity activities by accelerometry [37, 38], some studies indicate that most children spend a large proportion of their awake time being active at low levels of intensity [39–41], especially girls [40]. Although it is beyond the scope of this article, we also examined the associations of light-intensity physical activity and total sedentary time with BMI z-scores and only found weak associations (*r* = 0.03 and *r* = 0.08, respectively). This is in line with current recommendations and previous research showing that MVPA, screen time and sleep duration are the components of the 24-h day that are more strongly associated with obesity and other health outcomes [2, 4, 11, 14]. The relationship between light-intensity physical activity and health outcomes is largely unknown, partly due to the difficulty of its quantification as many methods to measure physical activity are not precise enough to capture such activities [38]. Consequently, there are currently no specific recommendations for light-intensity activities or non-screen sedentary behaviors. Future research will help to better understand the role played by all components of movement in the 24-h period on various health outcomes.

Major strengths of this study include the large international sample, the rigorous standardization of measurement and data collection across all study sites, and the combined use of objective and self-reported methods to measure MVPA, screen time and sleep duration. The limitations lie in the cross-sectional study design precluding any cause-and-effect associations between meeting the 3 health related behaviors of the 24-h movement guidelines and adiposity, the possibility of residual confounding by unmeasured variables, the analysis of sleep duration without a measure of sleep quality, the use of a self-reported questionnaire to assess screen time, and the limited generalizability of our data to the whole population.

## Conclusions

Meeting the new Canadian 24-h movement guidelines was associated with lower odds ratios for obesity and lower BMI z-scores in an international sample of children from sites in all inhabited continents and with a wide range of human development. Meeting all three movement behavior recommendations resulted in the lowest odds ratios for obesity, while meeting two guidelines was better than meeting one, and meeting one was better than meeting none. Collectively, these results suggest that the whole day matters from a movement perspective and future interventions and messages should encourage an increase in MVPA, a reduction in recreational screen time, and an adequate sleep duration. Further research needs to assess the possible value-added of this more integrated, holistic movement behavior approach on health.

## Abbreviations

BMI: Body mass index
CI: Confidence interval
COSI: Childhood obesity surveillance initiative
ENERGY: EuropeaN Energy balance Research to prevent excessive weight Gain among Youth
HDI: Human development index
ISCOLE: International study of childhood obesity, lifestyle and the environment
MVPA: Moderate-to-vigorous physical activity
OR: Odds ratio
WHO: World Health Organization
